# Generating evidence on screening, diagnosis and management of non-communicable diseases during pregnancy; a scoping review of current gap and practice in India with a comparison of Asian context

**DOI:** 10.1371/journal.pone.0244136

**Published:** 2021-02-01

**Authors:** Theophilus Lakiang, Sonali Abner Daniel, Kauma Kurian C., Minashree Horo, Shumayla Shumayla, Sunil Mehra

**Affiliations:** MAMTA Health Institute for Mother and Child, New Delhi, India; University of Mississippi Medical Center, UNITED STATES

## Abstract

**Background:**

Children born to high-risk pregnancies are more likely to experience adverse health outcomes later in life. As estimated, 15% of all pregnancies are at risk of various life-threatening conditions leading to adverse maternal and foetal outcomes. Millennium Development Goal resulted in the global reduction of maternal death from 390,000 to 275000 in 1990–2015). Similarly, to keep this momentum, the current United Nations Sustainable Development Goal (SDG: 3.1) aims at reducing the global maternal mortality ratio to less than 70 per 100,000 live births by 2030, and this can be achieved by addressing high-risk pregnancy contributing to significant mortality and morbidity. In India, gestational diabetes, gestational hypertension, and gestational hypothyroidism were identified as factors contributing to the high-risk pregnancy. This review summarises the commonly used approach for screening, diagnosis, and management of these conditions in the Asian population. It draws a comparison with the current protocols and guidelines in the Indian setting.

**Methods:**

Electronic search in PubMed and Google Scholar, reference snowballing, and review of current guidelines and protocols were done between January 2010 to October 2019. Published studies reporting Screening, diagnosis, and management of these conditions were included. Articles selected were then screened, appraised for quality, extract relevant data, and synthesised.

**Results:**

Screening, diagnosis, and management of these three conditions vary and no single universally accepted criteria for diagnosis and management exist to date. In India, national guidelines available have not been evaluated for feasibility of implementation at the community level. There are no national guidelines for PIH diagnosis and management despite the increasing burden and contribution to maternal and perinatal morbidity and mortality. Criteria for diagnosis and management of gestational diabetes, gestational hypertension, and gestational hypothyroidism varies but overall early screening for predicting risk, as reported from majority of the articles, were effective in minimizing maternal and foetal outcome.

**Conclusion:**

Existing National guidelines for Screening, Diagnosis, and Management of Gestational Diabetes Mellitus (2018) and Gestational Hypothyroidism (2014) need to be contextualized and modified based on the need of the local population for effective treatment. Findings from this review show that early screening for predicting risk to be an effective preventive strategy. However, reports related to a definitive diagnosis and medical management were heterogeneous.

## Introduction

Non-communicable diseases (NCDs) make up a considerable health burden in India, requiring a comprehensive approach of preventive, curative, and rehabilitative services and inter-sectoral coherence [1]. Women with diabetes, cancer, obesity, hypertensive pregnancy disorders, and several other NCDs are at a higher risk of developing childbirth-related complications, and so are their newborn child. Children born to mothers with NCDs are more likely to experience adverse health outcomes later in life [2–6]. An estimated rate of 15% of all pregnancies is at risk of suffering from various life-threatening conditions leading to fatal maternal and foetal that can happen anytime during the perinatal period. World Health Organization reported that close to 830 women died because of complicated pregnancy and labour. High-risk pregnancy contributed to about 70–80% of perinatal mortality and morbidity [7, 8]. Millennium Development Goal (MDG) resulted in the global reduction of maternal death from 390,000 to 275000 in the span of twenty-five years (1990–2015). Similarly, keeping the dynamic from the MDG, the current United Nation Sustainable Development Goal (SDG: 3.1) aims at reducing the global maternal mortality ratio to less than 70 per 100,000 live births by 2030, and this can be achieved by addressing high-risk pregnancy contributing to significant mortality and morbidity [9] and a more holistic approach to improving maternal health is needed, which includes addressing the global burden of NCDs contributing to maternal mortality and morbidity [10].

In India, although the reduction of maternal and infant mortality in recent years was substantial, an estimated number of 44000 pregnant women and 6.6 lakh neonates died each year because of preventable factors warranted for early screening and detection [11]. Ministry of Health and Family Welfare (MoHFW) of the government of India initiated the Pradhan Mantri Surakshit Matritva Abhiyan (PMSMA) programs aiming at providing universal, comprehensive and quality free of cost antenatal care. Pregnancy Induced Hypertension (PIH), Gestational Diabetes Mellitus (GDM), and gestational hypothyroidism (GH) were identified as factors contributing to a high-risk pregnancy [8]. MoHFW also published guidelines for diagnosis and management of GDM and GH but implementing the guidelines needs to be evaluated for its applicability and feasibility of implementing in the local population. Universal screening for high-risk pregnancy in NCD context is still a relatively new concept and needs further exploration for improving coverage, efficacy, and delivery [12, 13]. Research and planning in terms of programmatic evidence, best practices, risk factors, field screening methods, primary care models, prevention, and lifestyle modifications need to be adopted for promoting better care for high-risk pregnancies. Considering several factors and the knowledge-practice gap in the screening, diagnosis, and management of high-risk pregnancy, this rapid scoping review was planned to summarise the existing approach to screening, diagnosis, and management of GDM, PIH, and GH in the Asian population and draw a comparison with the current protocols and national guidelines in the Indian setting.

## Methodology

This review was developed and reported following Preferred Reporting Items for Systematic Review and Meta-analysis extension for Scoping Review (PRISMA-ScR) Guidelines [14]. This scoping review was not registered in any review registry.

### Criteria for considering studies for this review

**Types of studies:** Published studies (between January 2010 to October 2019) reporting screening, diagnosis, and management of PIH, GDM, and GH and report of public health program, policies, and guidelines. All reviews, meta-analyses, letters, editorials, commentaries, conference papers and report published in a language other than English were excluded. **Type of participants:** Pregnant women of any Gestational Age. **Types of intervention:** Screening, diagnosis, and management of GDM, PIH, GH for improving maternal and foetal outcome. **Outcome:** Diagnostic accuracy, common management modalities, efficacy, safety, adverse reaction, and maternal and fetal status. **Location:** Studies reported from Bangladesh, India, Indonesia, Maldives, Nepal, Pakistan, Singapore, and Sri Lanka were considered.

### Search methods

An Electronic search was performed in PubMed, manual search from Google Scholar, snowballing of relevant articles, and review of national program/policy/guidelines.

### Selection of studies

**Fig 1:**
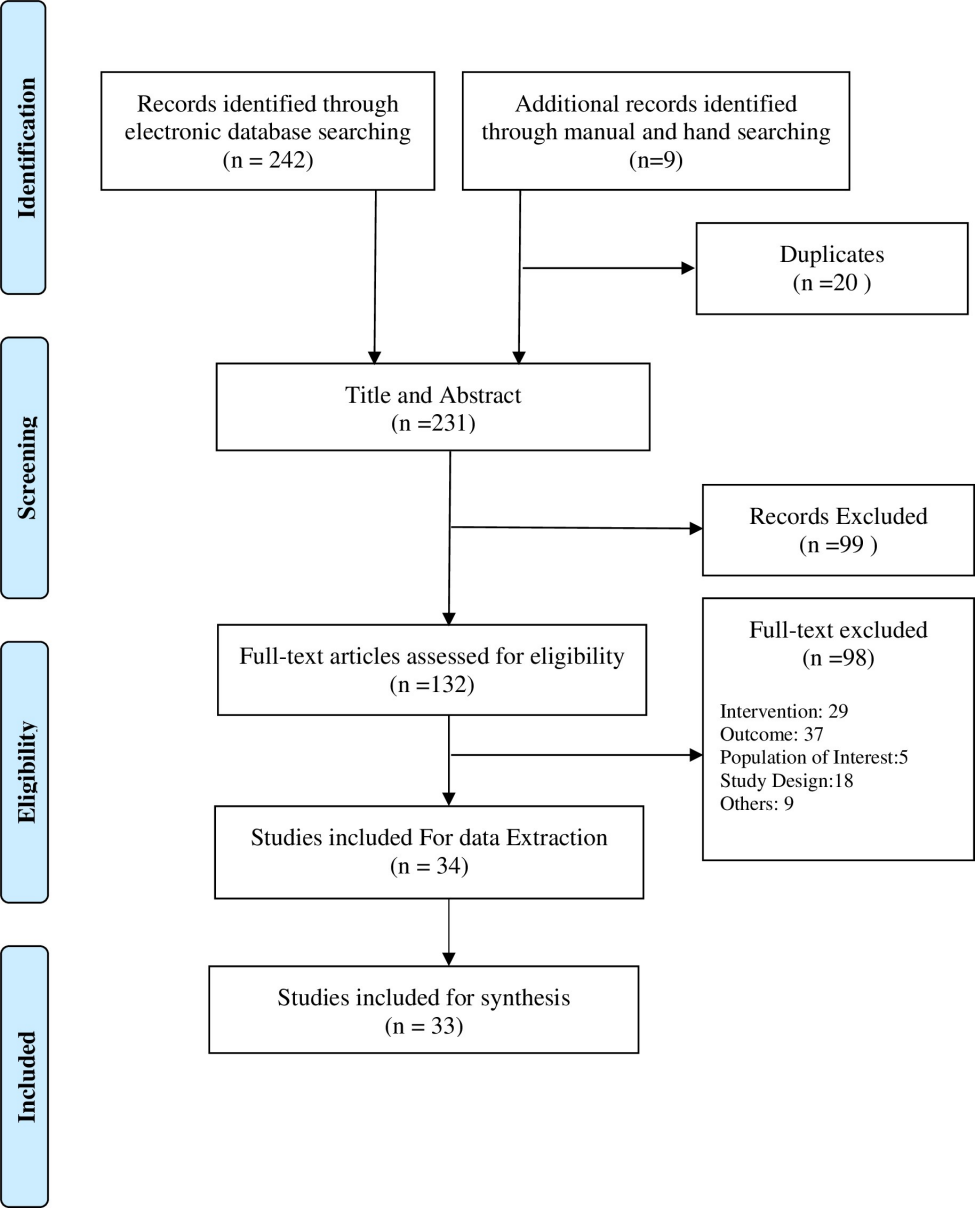
PRISMA chart.

All articles were subjected to screening for inclusion against a set of predetermined criteria. Title and abstracts were screened independently by two reviewers, and studies were taking forward only when both reviewers reached a mutual agreement for inclusion. A third reviewer arbitrated any disagreement between the reviewers to reach a consensus. For full-text screening, only those studies approved by both authors were included in the review. The rationale for exclusion was provided for all excluded studies. A PRISMA chart illustrating search and screening results is depicted in Fig 1.

### Data extraction and synthesis

Data were extracted by one reviewer and verified by two experts using a standardised, pre-tested data extraction form. A thematic narrative synthesis of included articles was done for summarizing each screening and diagnostic criteria and management protocol with the associated maternal-fetal outcome.

## Results

### Search result

242 articles were identified from PubMed search and 9 from google scholar and reference snowballing, and 231 articles were screened on title and abstract after removing 20 duplicates in which 99 articles were excluded leaving 132 articles for full-text screening. On full-text screening, 98 articles were excluded leaving 34 articles for data extraction and quality appraisal, and 33 studies were included for narrative synthesis. Screening process and reason for exclusion detailed in Fig 1. Data extracted from included studies are included in supplementary tables.

### Quality appraisal

The appraisal was done for all the papers included after the full-text screening. The quality of evidence of each article included in the review was assessed independently by two reviewers using the Joanna Briggs Institute Quality Appraisal tool [13]. The overall quality was good as the majority of the articles sufficed the criteria in the checklist. There was no disagreement between the two reviewers, and a total of 33 papers were finally included in this review for narrative synthesis.

### Characteristics of included articles

A total of 34 papers have been included in this review, and the study characteristics of all the 33 papers were given in Table 1. These ten studies were analytical cross-sectional studies, five cohort studies, four case-control studies, three quasi-experimental studies, three diagnostic test accuracy studies, two randomized controlled trials, one qualitative study, and one prevalence study. The majority (79%) of the studies were reported from India (27) and two from Nepal, two from Pakistan, one from Bangladesh, one from Sri Lanka, and one from Indonesia.

**Table 1:** Characteristics of the included studies.

Study	Study Design	Objectives	Population Characteristics	Setting
**Shrestha (2011) [17]**	Prospective and analytical study	To explore the feasibility of 50g GCT for determining the incidence of GDM and observing the maternal and foetal outcome of those with elevated GCT level	Sample Size: 1598	Out-patient department of Dhulikhel Hospital, Nepal
			Gestational Age: 24–28	
**Dahanayaka (2014) [23]**	Community based cross sectional study	To evaluate the present risk factor based approach used in the diagnosis of GDM in Sri Lanka, drawing comparison from the new guidelines proposed by the IADPSG.	Sample Size: 405	Anuradhapura district: Nuwaragampalatha East, Nuwaragampalatha Central and Kekirawa, Sri Lanka
			Gestational Age: 24–28	
**Gopalakrishnan (2015) [19]**	A cross sectional study	To analyse the prevalence and risk factors associated with GDM using the IADPSG criteria in India	Sample Size: 332	Antenatal clinics of urban hospitals; Queen Mary Hospital; Dr. Ram Manohar Lohia Institute of Medical Sciences and Sanjay Gandhi Postgraduate Institute of Medical Sciences, Lucknow, UP, India
			Gestational Age: 24–28	
**Rajput (2012) [24]**	Evaluation of Diagnostic Test Accuracy	To evaluate the utility of HbA1c in combination with OGTT GDM diagnosis.	Sample Size: 607	Antenatal Clinic of Post Graduate Institute of Medical Sciences, Rohtak, Haryana, India
			Gestational Age: 24–28	
**Khan (2019) [25]**	Cross-sectional analysis	To evaluate glucose tolerance patterns of pregnant women subjected to 2-H OGTT for comparing fasting, 1-H, 2-H post-glucose load results, HbA1c, the sum of all glucose readings with and without GDM using IADPSG criteria	Sample Size: 280	Departments of Pathology and Gynaecology, PNS Hafeez, Islamabad, Pakistan
**Mohan (2014) [18]**	A Cross Sectional Study	To compare the sensitivity and specificity of the non-fasting OGTT recommended by DIPSI with the WHO 1999 and IADPSG criteria for diagnosing GDM.	Sample Size: 1400	20 antenatal clinic from Chennai urban and Kanchipuram rural primary health centres, Tamil Nadu, India
**Bhavadharini (2016) [21]**	Evaluation of Diagnostic Test Accuracy	To compare CBG vs. VPG estimation using the IADPSG criteria as the gold standard for diagnosis of GDM	Sample Size: 1031	Urban: 9 health centres in Chennai Rural: 11 centres in Kanchipuram district, Tamil Nadu, India
**Singh (2017) [26]**	Prospective non-randomized intervention.	To evaluate the safety and efficacy of Metformin in the management of Gestational diabetes.	Sample Size: 2797	Antenatal clinic at Department of Obstetrics and Gynaecology, AIIMS, New Delhi, India
**Uma (2016) [27]**	Prospective Study	Impact of a structured MOC designed for limited resource setups for improving pregnancy outcomes of Asian Indian women with GDM.	Sample Size: 1124	Six maternity centres offering antenatal services in Chennai, India
			Gestational Age: <28	
**Seshiah Veeraswamy (2016)[20]**	Multi-centre, observational study	To assess the frequency of occurrence of glucose intolerance in the early gestational period.	Sample Size: 11785	131 prenatal clinics of private and public institutions across India
**Nielsen (2016)[22]**	The cohort study	To examine the role of potential risk factors for Hyperglycaemia in pregnancy: GDM and DIP, and investigate risk factor(s) for predicting Hyperglycaemia in pregnancy among women screened for GDM	All Pregnant Women	Urban: private diabetes centre Chennai, Semi-urban: government maternity health centre in Saidapet Rural: government primary health centre in Thiruvallur district), India
			n = 4053	
**Kamat (2011) [30]**	Prospective longitudinal study	To evaluate the possibility of using FMD as a non-invasive test for predicting PIH in high risk pregnancy.	Sample Size: 85	Postgraduate Institute of Medical Education and Research, Chandigarh, India
			Gestational Age: 18–24	
**Pal (2011) [42]**	Analysis of hospital records	To analyze the incidence of eclampsia, evaluate existing treatment protocols	Sample Size: 5991 records	Burdwan Medical College, a tertiary care hospital, India
**Singh (2013)[39]**	Prospective observational study	To evaluate microalbuminuria at mid-pregnancy using the ACR as a predictor of pre-eclampsia	Sample Size: 144	Department of Obstetrics and Gynaecology, and Pathology, CSM Medical University, Lucknow, India
			Gestational Age: 24–28	
**Mallick (2014)[32]**	Prospective analytical study	To evaluate the combined value of serum βhCG levels and serum lipids during 14 to 20 weeks of pregnancy with subsequent PIH/preeclampsia	Sample Size: 100	Department of Obstetrics & Gynaecology, Silchar Medical College & Hospital, Assam, India
			Gestational Age:14–20	
**Shahid (2019)[36]**	Prospective study	To assess the prognostic ability of sTWEAK in a high risk pregnancy	Sample Size: 63	Obstetrical clinics of Jinnah Post Graduate Medical Centre; Aga Khan University, Karachi, Pakistan
			Gestational Age: 12- Term	
**Kumar (2016) [33]**	A prospective cohort study	To evaluate the values of PAPP-A, hCG, maternal age, BMI, parity, MAP, and uterine-artery Doppler findings to predict PIH in low-risk pregnancy.	Sample Size: 2190	The Prenatal outpatient department of Lady Hardinge Medical College, New Delhi, India
			Gestational Age: 11 to 13 weeks+, 6 days of pregnancy.	
**Saraswathy (2016) [37]**	Cases Control Design	Evaluate the association of hypermethylated RASSF1A gene for the quantification of cffDNA and its correlation with pp13 and PAPP-A among those who have been clinically diagnosed with preeclampsia.	Sample Size: 719	Antenatal centre of a tertiary care hospital, Delhi, India
			Gestational Age: 21–40	
**Verma (2016) [34]**	Retrospective study	evaluate the diagnostic accuracy of noninvasive PE screening test at 11–13 weeks+6 days of gestation for predicting risks of early and late onset PE.	Sample Size: 615	A tertiary care hospital, New Delhi (Sir Ganga Ram Hospital), India
			Gestational Age: 11–13 weeks+6 days of pregnancy.	
**Agarwal (2017) [35]**	A nested case control model	To investigate a combined clinical MAP, Ut-PI, and PLGF model for predicting PE in the late 1^st^ trimester.	Sample Size: 291	A tertiary care Hospital, India
			Gestational Age: 11–14	
**Niraula (2017) [38]**	A hospital based comparative cross-sectional study	To assess and compare serum uric acid, creatinine, and urea between PIH and normotensive pregnancy and to determine the diagnostic efficiency of uric acid as a predictor for severity in PIH.	Sample Size: 90	BPKIHS, Dharan, Nepal
			Gestational Age: ≥24–36	
**Khanam (2018) [74]**	Retrospective Cohort Study Design.	To assess the effects of different durations of low-dose calcium supplement during pregnancy on the incidence of PIH.	Sample Size: 11387	10 sub districts of Mymensingh, Rangpur, Lamonirhat, and Kurigram, Bangladesh
			Gestational Age:3 to 9 months	
**Leigh (2018) [44]**	Cost-consequence analysis of the published study.	To compare the effectiveness between trans cervical Foley catheter and oral misoprostol tablets to induce labour among hypertensive women.	Sample Size: 602	Two public hospitals in Nagpur, India
			Gestational Age: 29–42	
**Nathan (2018) [75]**	Qualitative evaluation	To evaluate the usability, feasibility, and acceptability of the CRADLE VSA	Sample Size: 20	CHC and PHC in multiple areas in India
			Nurses; ANMs and ASHAs	
**Pedrana (2019) [70]**	Quasi-experimental pre-post evaluation	To evaluate the impact of the EMAS program on referral system services for effective management of complicated pregnancy.	Sample Size: 28 340 maternal referral cases	400 hospitals and CHC (puskesmas) in 30 districts and cities in six provinces. (North Sumatra, Banten, West Java, Central Java, East Java, and South Sulawesi), Indonesia
**Malhotra (2018) [31]**	Screening test evaluation	Predicting gestational hypertension/ preeclampsia by assessing endothelial dysfunction	Normal Primigravida of all ages visiting the ANC clinic in the first trimester.	Government Hospital, Chandigarh, India
**Kumar (2014) [41]**	Prospective cohort study	To evaluate the management prescription behaviour in patients with severe preeclampsia and eclampsia and the associated maternal and foetal outcome	Sample Size: Out of 198 screened, 164 included in the study	Obstetrics and Gynaecological department of Sir Sundar Lal Hospital of Institute of Medical Sciences, Banaras Hindu University, Varanasi, India
**Keepanasseril (2017) [43]**	A randomised open-label, parallel arm, non-inferiority Trail	To compare the efficacy of two drug regimens; the Dhaka regime vs. loading dose only regime as prophylaxis for seizures in severe pre-eclamptic cases	Sample Size: 402	Department of Obstetrics and Gynaecology of Jawaharlal Institute of Medical Education and Research, Pondicherry, India
			Gestational Age: 34–36	
**Prabahar (2019) [40]**	A Hospital based Retrospective observational study	To evaluate the efficacy and safety of administering anti -hypertensive drugs in gestational hypertension cases admitted to a tertiary care hospital	Sample Size: 200 records	Medical Records Department of a Gynaecological hospital, India
**Mundle (2017) [45]**	Multi-centric open labelled RCT	Comparing relative risks and benefits of low-dose oral misoprostol tablets and transcervical Foley catheterisation in low resource settings	Pregnant women ≥18 years of age, ≥20 weeks GA with a live foetus, planned for vaginal induction of labour because of pre-eclampsia or hypertension	2 government hospital in Nagpur, India
			Foley catheter n = 300	
			Oral misoprostol n = 302	
**Gayathri (2009) [47]**	A clinic based cross sectional study	To examine the prevalence of gestational subclinical hypothyroidism thyroid autoimmunity in pregnant women in the euthyroid state	Sample Size: 500	Institute of Obstetrics and Gynaecology, Egmore, and RSRM hospital; Chennai, India
			Gestational Age: ≤ 36	
**Nambiar (2011) [48]**	Cohort Design	To evaluate the effect of thyroid dysfunction on pregnancy outcomes	Sample Size: 483	The antenatal clinic of a tertiary referral centre, Mumbai, India
			Gestational Age: 8–12	
**Sahu (2010) [49]**	A prospective evaluation Study	To assess the impact of thyroid dysfunction on maternal outcome	Sample Size:633	Antenatal clinic of a tertiary care teaching hospital: KGMU, Lucknow and AIIMS, New Delhi, India
			Gestational Age:13–26	
**Ajmani (2014) [50]**	Prevalence Study	To evaluate thyroid dysfunction in normal pregnancy and the impact on maternal and foetal outcome	Sample Size: 400	Department of obstetrics and gynaecology at Kasturba Hospital, India
			Gestational Age:13–26	

### Summary of included articles

We identified 11 studies related to GDM, 18 related to PIH, and 4 related to GH. Studies identified for GDM and PIH included in this review were mostly related to screening and diagnosis. In contrast, all the papers identified for GH were prevalence studies and vaguely mentioned diagnosis and treatment protocols followed. Table 2 provides an overview of the summary of findings for each article included in the narrative synthesis.

**Table 2:** Summary of findings.

Study Name	Screening/Management	Result	Concluding remarks
**Shrestha (2011) [17]**	50 g GCT followed by 100 g OGTT for VBG ≥140mg/dl and diagnosis of GDM by Carpenter Coustan Criteria	198 women were subjected to 100g OGTT, and 12 women had GDM given a diagnostic yield of 6.06%.	Only one-fifth of the population would need to undergo the OGTT if the cut-off value is 140mg/dl.
**Dahanayaka (2014) [23]**	Combined Risk factor based +WHO criteria Vs. IADPSG	Prevalence of GDM was reported to be 8.9% as per IADPSG criteria and 7.2% based on a Risk approach and WHO criteria, and the overall reported prevalence was 10.6% (n = 43)	Risk based approach would have detected only 22 GDM patients according to IADPSG criteria, missing 14 cases (38.9%) who did not suffice the risk factors based approach
		Out of 29 who fulfilled WHO criteria, only one had an abnormal FBG, but 28 had abnormal 2H-PPBS.	
		170 (42.0%) participants had at least one eligibility factor/risk factor for OGTT at 24–28 weeks, and of these WHO-FPG criteria was satisfied in only one while 2H-PPBS in 21 at risk women.	
**Gopalakrishnan (2015) [19]**	Diagnosis of GDM as per the newer IADPSG criteria	GDM was diagnosed in 41.9% of women, and of these, 91.4% had abnormal FBG values. When the previous WHO criteria were applied, the study reported a prevalence of 17.5%	A large proportion of participants would be classified as GDM using the IADPSG criteria as compared to the WHO criteria
**Rajput (2012) [24]**	HbA1c, in combination with OGTT using criteria American Diabetes Association (ADA) and IADPSG for diagnosis of GDM.	HbA1c as per ADA at a cut-off value of ≥5.95% had a sensitivity of 28.6% and specificity of 97.2% in while an HbA1c cut-off value of ≥5.45% had a sensitivity of 85.7% and specificity of 61.1%.	HbA1c would have obviated an OGTT in 61.8% of women using HbA1c≥5.95% as the cut-off with high specificity.
		GDM defined by IADPSG criteria at HbA1c cut-off value of ≥5.95% had a sensitivity of 11.9% and specificity of 97.1% and HbA1c cut-off value of ≥5.25% had a sensitivity of 83.1% and specificity of 40.5%	
**Khan (2019) [25]**	Comparing the Diagnosis of GDM using GTT vs. HbA1c	The mean FBG at fasting, at 1-hour and 2-hour for both diagnosed and non-diagnosed GDM, were; 5.37±1.59 vs. 4.10±0.53; 10.67±3.22 vs. 6.71±1.50; and 8.88±3.41 vs. 5.57±1.10, respectively. The study reported that adding all glucose estimation values produced and ROC with AUC = 0.962 (95% CI: 0.935–0.988) and the lowest was for HbA1c; AUC = 0.668	The highest levels of HbA1c were in subjects who demonstrated a delayed 2-hour post-glucose load peak. The additive sum of glucose readings showed the highest AUC.
**Mohan (2014) [18]**	Diagnostic accuracy if DIPSI Criteria compared to WHO 1999 criteria and IADPSG	DIPSI vs WHO: Sensitivity/Specificity of 27.7%/97.7% at 140 mg/dl cut-off and a sensitivity/Specificity of 72.3%/68.6% and 85.5%/47.7% when cut off value is reduced to 110 mg/dl and 100 mg/dl respectively.	DIPSI guidelines can be used in resource limited setup with a two-step procedure; non-fasting 50g OGCT for initial screening and OGTT definitive diagnosis for those screened positive
		DIPSI vs IADPSG: Sensitivity/Specificity at 140 mg/dl cut-off was 22.6%/97.8% and a sensitivity/Specificity of 65.1%/69% and 78.3%/47.5% when cut off value is reduced to 110 mg/dl and 100 mg/dl respectively.	
**Bhavadharini (2016) [21]**	Capillary blood glucose (CBG) vs. VBG using IADPSG criteria for the diagnosis of GDM.	2H-CBG: Sensitivity and specificity of 2H CBG with IADPSG as the gold standard was 70.8%/63% at 126 mg/dl cut-off and Sensitivity/Specificity 78.3%/53.1% and 92.5%/33.7% at 120 mg/dl and 110 mg/dl cut-off, respectively.	CBG can be used as an initial screening strategy in resource limited setup followed by definitive diagnosis OGTT with VPG estimation.
		FCBG: at cut-off value of 80 mg/dl, 75 mg/dl and 70 mg/dl the reported Sensitivity/Specificity were 67.9%/58.5%, 79.3%/36.9% and 92.5%/16.1% respectively.	
**Singh (2017) [26]**	Efficacy of metformin 500 mg, OD administered 15 minutes before breakfast among those with ≥2 abnormal VBG values; FBG >95 mg/dl and 2hour PPBG value >120 mg/dl) post Medical Nutrition Therapy Induced labor at 38 weeks.	93.8% (60) of women reported having achieved the desired blood sugar level with a Mean dosage of 1226.5±397.1 mg, and only one requires insulin therapy.	Metformin may be a safer and effective oral hypoglycemic agent specifically useful in a limited resource setting
		Minor side effects were reported in 3.1% (2) of women, which was spontaneously resolved.	
		The reported Mean birthweight was 2753.5 ± 565.4g with 14.1% (9) SGA neonates and 15.6% (10) large for gestational age.	
		About 14% (9) of neonates require NICU care.	
**Uma (2016) [27]**	Structured model of care (MOC)- which included medical nutrition therapy (MNT), regular physical activity (PA), intensive counselling, regular blood glucose monitoring, and insulin therapy as indicated.	Follow up assessment: mean FBG of 86 ± 18 mg/dL; 1-h PPBG 122 ± 20 mg/dL; and 2-h, 114 ± 28 mg/dL, 84.4% (179) were treated with MNT and exercise alone.	Management of GDM with focused counselling can be effective in minimizing the need for insulin therapy.
		Only 15.6% (33) required insulin at some point in time during follow up.	Effective in bringing down maternal and neonatal complications.
		Maternal and fetal complications between GDM and non GDM cases were not statistically significant except for neonatal hypoglycemia (3.3% vs. 0.83%, P value <0.000)	
**Seshiah Veeraswamy (2016) [20]**	Single Step Procedure (SSP)- administration of 75 goral glucose irrespective of the last meal timing and diagnose GDM with 2H-VBG value ≥7.8 mmol/L (7.8 mmol/L)-WHO 2013	740 (8%) pregnant women were diagnosed with GDM, and 31.5% of these were detected in the first trimester, 43.2% in the second trimester, and 25.3% in the third trimester.	The need for early screening and diagnosis was strongly recommended since a third of every case was detected within the 2^nd^ trimester.
		About 27% of those with GDM in the 1^st^ trimester reported a history of fetal loss in their previous pregnancy, suspecting that they might also be present with a similar condition in the past pregnancy	
**Nielsen (2016) [22]**	Risk factor based approach for predicting Hypertension in pregnancy.	The ROC for age: 25 years (69.3% sensitivity and 57.7% specificity) best cut-off value.	Screening women with one or more risk factors could identify 86.1% of all the HIP cases and avoid the need for 32.6% of OGTTs. Still, Sensitivity and specificity were not reliable when replicated in other settings. Hence, universal screening still is the best available approach.
		ROC for BMI: 22.3kg/m2 for BMI (66.6% sensitivity and 57.4% specificity) best cutoff value.	
		The best yielding sensitivity and specificity of risk factors were for anyone risk factor 86.1% sensitivity and 37.1% specificity; and two or more risk factors 55.9% sensitivity and 73.4% specificity.	
		Overall, age ≥ 25 years and BMI ≥ 22.3 correctly identified two thirds of women with the HIP.	
**Kamat (2011) [30]**	Flow mediated vasodilatation (FMD) of the brachial artery as a non-invasive test for predicting pregnancy induced hypertension in high risk pregnancy.	Cutoff value which gives most reasonable sensitivity and specificity for FMD1 was 8.7% (Sensitivity:88%, Specificity:93%, PPV:84% and NPV:94.8%) and FMD2 was 9.5% (Sensitivity:88%, Specificity:98.21%, PPV:95.65% and NPV:94.83%).	FMD of the brachial artery for predicting PIH in pregnancy as early as the nine weeks of gestation way before the manifestation can be clinically detectable is an effective approach, but further larger scale evaluation is required.
		About 62% of those with FMD <9% developed PIH and 16% for those with FMD of >10%.	
**Pal (2011) [42]**	Menon regime (Lytic cocktail)	Recurrence of convulsion: lowest among those treated with inj. magnesium sulphate (5.7%) as compared to phenytoin sodium (10.9%), lytic cocktail (32.8%), and diazepam (40.3%).	The study concluded that the use of magnesium sulphate access to appropriate emergency obstetrics care facilities help in lowering maternal and foetal mortality rate in the institution.
		Induction delivery interval: lowest in Phenytoin	
	Phenytoin sodium injection	Sodium treated cases 12.75±2.4 hrs. and highest in Diazepam 22.23±3.2	
	Diazepam		
	Magnesium sulphate Injection	Prevalence of LSCS was highest among those treated with MgSO4 (27.55%) and phenytoin sodium (22.63%).	

		Perinatal mortality: lowest with MgSO4 Monotherapy (22.4%) and highest with Phenytoin Sodium (32.5%).	
		Prevalence of LBW: lowest in MgSO4 treated cases 15.68% as monotherapy	
**Singh (2013) [39]**	Screening of PE via evaluating microalbuminuria at mid pregnancy, using the ACR	22.9% microalbuminuria Was observed, and of these, 36% developed pre-eclampsia. Mean ACR was significantly higher among severe pre-eclampsia cases (123.9±59.7 mg/g)	Since microalbuminuria was observed in 22.9% of low risk pregnancy; microalbuminuria can potentially be an effective predictor for pre-eclampsia, and ACR is the optimal tool for detecting microalbuminuria
		ACR accuracy for predicting Pre-eclampsia; sensitivity of 92.3%, specificity of 83.9%, PPV of was 36.7%, and NPV of 99.09%.	
**Mallick (2014) [32]**	Screening of pregnancy induced hypertension	Prevalence of PIH/Pre-eclampsia increases with an increased in serum βhCG	Those with high serum βhCG and impaired lipid profile in 2nd trimester are more prone to developing PIH and suggesting that these variables can be used for predicting PIH
		Mean serum βhCG, TC and TG were significantly higher in those who developed PIH/PE	
		Odds of developing PIH/PE increases with increased age and serum βhCG, TC, TG, and VLDL level.	
**Shahid (2019) [36]**	Screening of pregnancy induced hypertension	Baseline sTWEAK value was 4.03±0.37 ng/dl and subsequently reduced to 1.93±0.23 mg/dl at follow up and is significantly lower than normotensive high risk and negative control group (p<0.001).	A reduced PlGF level along with reduced sTWEAK levels in early pregnancy reported in this study, indicating its link to PE pathogenesis
		High risk group that developed PE/PIH have a significantly lower PIGF value	
**Kumar (2016) [33]**	The role of PAPP-A, free β-hCG, maternal age, BMI, parity, MAP, and uterine-artery Doppler to predict PIH in low risk pregnancy	Combined markers: sensitivity, specificity, positive predictive value, and negative predictive value of 76%, 80%, 31%, and 93%, respectively.	BMI and MAP alone or in combination with other markers can be used as a screening strategy in a limited resource setting.
		BMI + mean: sensitivity and specificity of 52% and 80%.	
**Saraswathy (2016) [37]**	Hypermethylated *RASSF1A* gene to quantify cffDNA and assess its relationship with pp-13, PAPP-A, and urine proteins.	Mean hypermethylated RASSF1A, pp-13, PAPP-A, and urine protein concentrations were significantly higher among cases as compared to controls (P < 0.001).	Findings from the ROC curve and Multivariate Pearson’s correlation analysis reflects that hypermethylated RASSF1A can be strongly recommended as a reliable approach for pre-eclampsia diagnosis.
		Mean concentrations pp-13 and PAPP-A showed an increasing pattern with GA (from 21 to 40 weeks).	
		pp-13 (r = 0.697, P < 0.001) and PAPP-A (r = 0.833, P < 0.001) shows a strong and significant positive correlation with hypermethylated RASSF1A	
**Verma (2016) [34]**	The combined effect of multiple variables: serum PLGF, PAPP-A, maternal age, parity, ethnicity, MAP, BMI, Ut-PI, and previous history of PE or hypertension (HT) for predicting of risks for early and late onset PE	Early onset: sensitivity/specificity was 44.4% 100% with a reported accuracy of 99.2%.	The NPV (>98%) reinforces the utility of a cost-effective non-invasive screening test for the early detection of PE.
		Late onset: sensitivity/specificity was 93.8%/97.7% with a reported accuracy of 97.5%.	
			This approach improves the accuracy and detection rate of both early and late onset PE.
		NPV was more than 100% for both early and late onset PE	
**Agarwal (2017) [35]**	Combined clinical (MAP), biophysical (Ut-PI), and biochemical (PLGF) model for predicting PE in the late first trimester.	PLGF alone: detection rate of 40% and 51% with 5% and 10% FPR respectively for pre-eclampsia whereas GH reported the lowest detection rate when PLGF was used alone, i.e., 16% and 21% with 5% and 10% FPR, respectively.	This indicates that a combined model (PLGF+MAP) is best suited for the prediction of preeclampsia rather than PLGF alone
		PLGF+MAP: AUC of 0.937 for PE and 0.833 for GH	
		PLGF+MAP+PI: AUC of 0. 965 for PE.	
**Niraula (2017) [38]**	Diagnostic efficiency of uric acid and creatinine as a predictive marker of severity in PIH	Serum uric acid: Sensitivity—79.07%, Specificity—71.19% Serum creatinine: Sensitivity e 62.75%, Specificity e 27.45%	Serum uric acid assessment is a convenient and efficient method for early prediction PIH.
**Leigh (2018) [44]**	Transcervical Foley catheter vs. oral misoprostol tablets to induce labour in hypertensive women.	Vaginal delivery ≤24 hrs. of induction were significantly higher in oral misoprostol (57%, 95% CI: 51.4–62.5%) compared to Foley catheter group (47%, 95% CI: 41.5–52.8%) with P 0.0162.	Oral misoprostol improves rates of vaginal delivery within 24 hrs. of induction.
		Vaginal delivery significantly higher in oral misoprostol (59.3%) as compared to the Foley catheter group (49.8%) with P = 0.0210.	
		The time interval from randomization to induction	
		commencement was four times higher for the Foley group (0.56 to 0.16 hours, P = 0.0004).	
		A reduction of 90 minutes from induction to delivery (14.35 vs. 12.85 hours, P = 0.0094) was observed in the oral misoprostol.	
**Nathan (2018) [75]**	Efficiency and convenience of CRADLE Vital Signs Alert, a novel device that measures blood pressure and pulse.	The device was easy to use in the community, detect hypertension accurately, and is comparable to other devices. The reading regarding hypertension drives the community health worker to take a clinical decision and thereby directing the patients to the health facility. Error alerts on the CRADLE device guided the health workers for the right way of eliciting and which was absent in the sphygmomanometer	Healthcare workers well accepted the device. The device motivated women to attend primary care and encouraged them to accept treatment and referral.
**Malhotra (2018) [31]**	Flow mediated vasodilatation (FMD) a predictor for gestational hypertension/pre-eclampsia	FMD significantly decreased from 1^st^ to 3^rd^ trimester	FMD is a potential non-invasive approach for predicting the development of GH/PE and can potentially be effective in predicting GH/PE in Primigravida females.
		A decreased of 57% was observed in those who developed Gestational hypertension/pre-eclampsia as compared to 39% who did not develop the condition (p < 0.01) was observed by the 3^rd^ trimester.	
		FMD at 3^rd^ trimester is effective in predicting development of condition with reported OR = 1.303; 95% CI 1.088–1.562; p = 0.004	
		ROC for 3^rd^ trimester FMD value with a cut-off of 3.6% generated a sensitivity/ of 69%/68% and a cut-off value of 3% shows a sensitivity of 80%, and at 2% cut-off sensitivity was 90%.	
**Pedrana (2019) [70]**	EMAS: facilitate timely and effective management of complicated pregnancy by forming referral network, monitoring performance standard, and implementation of an e-referral exchange system	The proportion of mothers referred to hospital due to severe pre-eclampsia /eclampsia who received magnesium sulphate (MgSO4) before referral significantly increases over time; 20% to 55% in Phase 1; 22% to 64% in Phase 2; and 32% to 60% in Phase 3 in the EMAS intervention setup.	EMAS program facilitates referral services by priming patients, health facility, and provider to effectively manage emergency obstetrics cases and minimizing delay in access to treatment.
**Kumar (2014) [41]**	Management of PIH	CCBs drug of choice was Nifedipine and amlodipine, and the preferred β-blocker was atenolol.	The high prescription rate of rapid acting sublingual Nifedipine prompt the author to recommend regular review of protocols for management of eclampsia and pre-eclampsia
		All eclampsia cases received Magnesium Sulphate (MgSO_4_), but the same was prescribed in only 5.8% of the pre-eclampsia cases.	
**Keepanasseril (2017) [43]**	Management of Preeclampsia	2.24% of women on prophylactic MgSO4 developed eclampsia; 1.49% in the low-dose Dhaka group and 2.98% in the loading dose only regime group.	The author concluded that a single loading dose only regime be an effective alternative to the standard 24-hour therapy.
		The mean birth weight of the babies was significantly lower in the ‘low dose Dhaka’ regime than those compared with the ‘loading dose only’ regime (1895.5 ± 49.5 g vs. 2053.0 ± 53.2 g, p = 0.031) group.	
**Prabahar (2019) [40]**	Efficacy and safety of administering anti -hypertensive drugs in gestational hypertension	51.5% (103) of the cases achieved controlled blood pressure with monotherapy, 39.5% (79) with dual drug therapy, and 3% (6) with triple drug therapy 6% (12) were unresponsive.	Nifedipine was reported to be a safe and effective anti-hypertensive for pregnancy induced hypertension.
		The percentage of preterm and late term deliveries was 19.5% (39) and 58% (116), respectively.	
		Pregnancy outcome reported: healthy mother & baby (65.5%), healthy mother but the sick baby (22%), sick mother but the healthy baby (1.5%), sick mother & baby (4%) and sick mother & dead baby (7%)	
**Mundle (2017) [45]**	Transcervical Foley catheter vs. oral misoprostol tablets for induction labour in hypertensive women in a low resource setting	Oral Misoprostol of 25 μg every 2 h for a maximum of 12 doses resulted in a mean interval time of 771 min (from the start of induction to birth) compared to 861 min for the Foley catheter group.	Thus, low-dose misoprostol (25 μg) can potentially be safe and effective for induction of labour for mild hypertensive pregnancy in a low resource setting. The study also reported the challenges of managing in the low resource setup; absence of infusion pumps is one and fluid overload due to IV oxytocin.
		Vaginal birth was also reported to be higher in the misoprostol (57·0% vs. 47·0%; absolute risk difference 10·0%, 95% CI 2·0–17·9) when compared to the Foley catheter group. A little over than half (52%) of the women in the oral misoprostol group requires oxytocin, whereas 81·6% of those in the Foley catheter group required oxytocin.	
**Gayathri (2009) [47]**	Estimate haemoglobin, urea, creatinine, random blood glucose, free T4, TSH, and TPO antibodies to identify hypothyroidism.	Subclinical hypothyroidism with TSH value cut-off of 5–10 mU/L was 2.8% (14), and cut-off of <2.3 mU/L increases the prevalence to 19%. TPO antibodies were positive among 57.1% (8) of hypothyroid (p = 0.012) and 7.1% (34) in euthyroid women. A significant increase in the prevalence of hypothyroidism with increased GA (c2 = 6.02, p = 0.014).	The study recommended thyroid screening to be included as a part of routine antenatal care to improve maternal and fetal outcomes.
**Nambiar (2011) [48]**	Diagnosing thyroid dysfunction	**Pregnant women with TSH 0.1–2 μIU/mL with thyroid autoimmunity (TAI)** reported to have a significant previous history of stillbirth (P = 0.042); rise in TSH by 0.78 μIU/mL as pregnancy advanced (P = 0.002); the titers of anti-TPO antibodies decreased progressively towards the last trimester by 85% (242 to 34 IU/mL) (P = 0.043), and miscarriage was 3 times more common (26.3% versus 7.35%).	In this study, 40% of the hypothyroid and 45% of TPOAb positive cases did not report any known high-risk characteristics, indicating that about 60% of the hypothyroid or TPOAb positive cases could have been missed by targeted case finding. Hence a universal screening is recommended
		**Pregnant women with TSH 2–4 μIU/mL without TAI** reported to have significantly increased TSH (by 0.25 μIU/mL; P = 0.029); decrease in FT3/FT4 (P = 0.025 and 0.033, resp.) at 6 months of pregnancy; 5.1% (2) had TSH >4 μIU/mL at 6 month follow up; and one still birth.	
		**Pregnant women with TSH > 4 μIU/mL** were reported to be older than other groups (P = 0.02).	
		**One patient with GD regularly followed up and required Propylthiouracil** throughout pregnancy in tapering doses; TRAb titers reduced at third trimester (27 IU/L to 14 IU/L) and had a normal delivery and normal thyroid hormone of the baby.	
		**Pregnant women with TSH 2–4 μIU/mL with TAI were reported to have been treated with thyroxine** with regular monitoring that led to the significant decline in FT3 and FT4 at the late half of the pregnancy.	
**Sahu (2010) [49]**	Prenatal thyroid dysfunction screening	Overt/clinical hypothyroidism (4.58%): high TSH+low-free T4.	This study reported a high prevalence of overt and subclinical hypothyroidism with associated adverse perinatal outcomes indicating the need for routine thyroid function screening for all pregnancies.
		Subclinical hypothyroidism (6.47%): high TSH in the presence of normal blood levels of thyroid hormone.	
		Overt hyperthyroidism (0.78%): Elevation in free T4 with an undetectable serum TSH.	
		Subclinical hyperthyroidism (0.94%): low serum TSH concentration in concert with normal thyroid hormone levels.	
**Ajmani (2014) [50]**	Thyroid function test	Euthyroid group (TSH 0.2–3.0 μIU/l), Subclinical hypothyroid (TSH >3.0 μIU/l), Overt hypothyroid (TSH >3.0 μIU/l with low Free T4 <0.8 ng/dl), Subclinical hyperthyroid (TSH <0.2 μIU/l with normal Free T4 0.8–2.0 ng/dl), Overt hyperthyroid (Free T4 (>2.0 ng/dl) with decreased TSH <0.2 μIU/l).	Findings from this study like a high maternal and adverse fetal outcome among subclinical and overt hypothyroidism and the fact that the prevalence of subclinical cases was the highest indicate the need for universal screening.
		The overall prevalence of thyroid dysfunction was 13.25% (53), and from these, 91% (48) were diagnosed with overt hypothyroidism (25%) and subclinical hypothyroidism (75%). The positive anti-TPO antibody was reported in 52% of the hypothyroid cases but none in hyperthyroid patients.	

### Gestational diabetes mellitus

GDM has been defined as "any degree of glucose intolerance with onset or first recognition during pregnancy.” GDM is a common disorder in pregnancy, but the lack of uniform screening or approach to diagnosis makes it challenging to assess its frequency in the differing population and estimate its global incidence. IADPSG/WHO 2013 criteria were currently recommended for screening and diagnosis [15]. Though the clinical value of management of GDM is questionable, recent evidence shows that effective screening and management of GDM help reduces associated adverse outcome. Management includes monitoring blood glucose level, dietary modification, exercise, and pharmacotherapy whenever required [16].

### Screening and diagnosis

Screening and diagnosis are crucial not only for detecting those with GDM but also in deciding on the treatment modalities. Estimation of PBG level is the basic approach to diagnosis; however, a single universally accepted cut-off still doesn’t exist. The most commonly used criteria were IADPSG, WHO 1999 and 2013, ADA, and DIPSI criteria. Estimation of PBG in pregnant women between 24 and 28 weeks of gestational age post 50g glucose load for GCT (glucose challenge test) followed by 100g OGTT for those with PBG ≥140mg/dl during the initial screening and diagnosed using the Carpenter Coustan criteria shows that 12.3% with 50g GCT value ≥140mg/dl but only 6.06% from these were diagnosed with GDM after a formal OGTT. This indicates that 140mg/dl cut-off is valuable as only one-fifth of the population would need to undergo OGTT, and the diagnostic yield was 6.06% [17]. Comparison of sensitivity and specificity of non-fasting OGTT recommended by DIPSI to that of WHO 1999 and IADPSG criteria reported that when pregnant women were exposed to 75 g OGTT (non-fasting) and 75-g OGTT (fasting) 2–3 days later from initial glucose load shows a diagnostic yield of 8% using the WHO-1999 criteria (but only 27.7% of these were also diagnosed with GDM using DIPSI and IADPSG), 4.2% using DIPSI criteria (52.2% of these were also diagnosed by IADPSG criteria) and 10.3% using IADPSG criteria (only 22.6% were diagnosed using the DIPSI criteria but 49.1% using the WHO criteria) while only 2% (22) of women were diagnosed with GDM by all three criteria. DIPSI was also reported to have a low sensitivity when compared to WHO (27.7%) and IADPSG criteria (22.6) at cut off point of 140 mg/dl indicating that DIPSI would miss out on 72.3% of cases diagnosed by WHO-1999 criteria and 77.4% those diagnosed with IADPSG criteria [18]. Similarly, a comparison between IADPSG and WHO 1999 criteria after 2-h 75-g OGTT in the fasting state reported a prevalence of GDM to be 41.9%, of which 91.4% had abnormal FBG value using the IADPSG criteria while only 17.5% was diagnosed using the WHO 1999 criteria indicating that a large proportion of participants would be classified as GDM using the IADPSG criteria as compared to the WHO criteria [19] and using WHO 2013 Single Step Procedure; estimation of PBG two hours after 75 g OGTT yields a prevalence of 8%; 31.5% were detected in the 1^st^ trimester, 43.2% in the 2^nd^ trimester and 25.3% in the 3rd trimester and about 27% of women diagnosed with GDM in the 1^st^ trimester reported a history of fetal loss in their previous pregnancy suspecting that they might also be present with a similar condition in the past pregnancy and almost a third of all the GDM cases were diagnosed at the 1^st^ trimester indicating the need for early screening and diagnosis [20]. One study [21] compared capillary blood glucose (CBG) estimation using a handheld glucose meter with the venous blood glucose (PBG) estimation using the IADPSG criteria as the gold standard for diagnosis, but CBG was found to be less efficient and might not be an appropriate approach but can be used for initial screening in resource-limited setup followed by definitive diagnosis with OGTT using PBG.

Risk factors based approach to screening and diagnosis were also reported in two studies. Factors such as age, BMI, and having only a mother or both parents with diabetes significantly increases the risk for HIP. The ROC curves for age and BMI showed the best cut-off values of 25 years for age (69.3% sensitivity and 57.7% specificity) and 22.3kg/m^2^ for BMI (66.6% sensitivity and 57.4% specificity). Overall, age ≥ 25 years and BMI ≥ 22.3 correctly identified two-thirds of women with Hyperglycaemia in pregnancy. Screening women with one or more risk factors could identify 86.1% of all the Hyperglycaemia in pregnancy cases and avoid the need OGTTs in 32.6% of cases. However, the sensitivity and specificity were not reliable when replicated in another setting. Hence, universal screening still is the best available approach [22]. Similarly, in Sri Lanka [23], the present risk-factor based approach was a recommended practice for the diagnosis of GDM. Pregnant women with one or more of these risk factors and 75 g OGTT results (WHO criteria) with fasting PBG ≥7 mmol/L and/or 2-hour PPBG ≥7.8 mmol/L can be diagnosed with GDM. PPBG after 2 hours was recommended as a screening test for those with any one of the risk factors, followed by 75g OGTT for those with PPBG of ≥7.2 mmol/L during the initial screening. Furthermore, those with OGTT of ≤7.2 mmol/L and with the presence of any one of the risk factors will again be subjected to 75g OGTT at 24–28 weeks of gestation. Prevalence of GDM was 8.9% as per IADPSG criteria and 7.2% based on WHO criteria with an overall prevalence of 10.6% (n = 43). Out of 29 mothers who fulfilled WHO criteria, only one had an abnormal FBG, but 28 had abnormal PPBG hour values. A total of 42.0% of participants had at least one eligibility factor/risk factors for OGTT at 24–28 weeks, and of these WHO FPG criteria were satisfied in only one woman and two hours PPBG in 21 women at risk mothers. This indicates that a risk-based approach would have detected only 22 GDM patients according to IADPSG criteria, missing 14 cases (38.9%) who did not suffice the risk factors based approach.

Estimation of glycated hemoglobin for diagnosis of GDM in combination with other blood glucose estimation was reported in two studies. HbA1c and PBG estimation at fasting, 1 hour and 2 hours following 75 g glucose load between 24–28 weeks of pregnancy using the ADA and IADPSG criteria and reporting that the value of HbA1c ranged from 4–6% with a statistically significant mean ± SD of 5.73 ± 0.34% and 5.34 ± 0.35% among women with and without GDM respectively with a sensitivity/specificity of 28.6%/97.2% at HbA1c cut-off value of ≥5.95% and 85.7%/61.1% at a cut-off value of ≥5.45% using the ADA criteria. Similarly, using the IADPSG criteria yielded a sensitivity/specificity of 11.9%/97.1% at HbA1c cut-off value of ≥5.95% and 83.1%/40.5% at a cut-off value of ≥5.25%. Considering ADA criteria, using OGTT for women with HBA1c level between 5.45 and 5.95% increase the sensitivity up to 85.7%, 2.8% of false positive and would rule out the need for OGTT in 61.8% of women included in this study. On the other hands, using the same method while considering IADPSG criteria; OGTT for women with HBA1c level between 5.25 and 5.95% will detect 83.1% of the case with 2.9% false positive and would rule out the need for OGTT in 39.66% of women included in this study. Hence, the study concluded GDM can be diagnosed using HBA1c ≥5.95% with high specificity and reduced the need for repeated OGTT (Rajput et al., 2012) [24]. Comparison of glucose tolerance patterns of pregnant women subjected to 2-H OGTT at fasting, 1-H, 2-H post-glucose load, HbA1c, the sum of all glucose readings among pregnant women with and without GDM using IADPSG criteria shows that the mean PBG at fasting, at 1-hour and 2-hour for both diagnosed and non-diagnosed GDM were; 5.37±1.59 vs. 4.10±0.53; 10.67±3.22 vs. 6.71±1.50; and 8.88±3.41 vs. 5.57±1.10 respectively and when adding all these glucose estimation values for diagnosing of GDM produced and ROC with AUC = 0.962 (95% CI: 0.935–0.988) however, AUC for HbA1c = 0.668. The study also reported a high level of HbA1c in subjects with delayed 2-hour post-glucose load peak (Khan 2019) [25].

### Common approach to management

Medical nutrition therapy (MNT) is the preferred and efficient approach to the management of GDM, with 71.3% (159) of women reported to have controlled blood sugar levels after two weeks of MNT and for those with uncontrolled blood glucose; 500 mg of Metformin also is as effective. Dosage was adjusted based on the blood glucose level, including regular maternal and fetal monitoring. Metformin monotherapy was found to be effective for optimizing the blood sugar level of GDM with a mean dosage of 1226.5±397.1 mg in 93.8% (60) of women, and only one requires insulin therapy as well. Minor side effects were reported in 3.1% (2) of women, which were spontaneously resolved in few days. The reported Mean birthweight was 2753.5 ± 565.4g, with 14.1% of neonates being small for gestational age and 15.6% large for gestational age. Apgar score taken at one minute was reported to be <7 3.1% of the neonates. About 14% of neonates require admission to a neonatal intensive care unit. The study also reported a high rate of caesarean section. This may be associated with the fact that pregnant women reporting to the tertiary hospital may have already had one or more risk factors. Overall, the study concluded that Metformin might be a safer and effective oral hypoglycemic agent specifically useful in a limited resource setting [26]. A structured model of care (MOC) designed for resource-constrained settings, which included screening for GDM followed by MNT for two weeks with FPBG and PPBG estimation every two weeks to monitor whether targeted blood sugar level is achieved or not. A combination of Insulin and MNT was given to those who failed to meet the targeted level. Dosage for insulin was determined by estimating FBG and PPBG every two weeks. Women were also provided with counselling and support by trained nutritionists, and health care professionals especially focused on lifestyle modification. About 15.6% (33) required insulin at some point in time during follow up, and 84.4% (179) were treated with MNT and exercise alone. Comparative analysis of maternal and fetal complications between GDM and non-GDM cases was not statistically significant except for neonatal hypoglycemia. However, this may not be a true representation since neonates born to non-GDM mothers are tested for hypoglycemia only if they manifest the condition or were admitted in the NICU. Similarly, the prevalence of pre-term deliveries, LBW, and Macrosomia among GDM was not statistically significant compared to non-GDM women. The study concluded that WINGS MOC was found to be effective in bringing down the rates of both maternal and neonatal complications to levels similar to those in pregnant women without GDM. The present study highlighted that the management of GDM through focused counselling towards lifestyle modification and tracing one’s physical and diet chart could be effective, given that 84.4% of the women in the MOC were treated with MNT and physical activity alone and only 15.6% required insulin therapy [27]. Similarly, a study in Nepal reported that all patients diagnosed with GDM were referred to a dietician, internist, and obstetrician for further management, put on 1800 kilocalorie diet insulin when indicated. For cases that required insulin therapy, pregnancy was terminated at 39 weeks of gestation [17].

### Gestational hypertension

Gestational hypertension is defined as a systolic BP of at least 140 mm Hg and/or a diastolic BP of at least 90 mm Hg on at least two occasions and 6 hours apart after the 20th week of gestation in women known to be normotensive before pregnancy and before 20 weeks’ gestation [28]. The screening and diagnosis may include blood pressure readings, urine analysis to check for protein, liver functioning tests, etc. Current practice for managing gestational hypertension includes anti-hypertensive drugs, regular monitoring of blood pressure, foetal growth monitoring, and management during labour [29].

### Screening and diagnosis

Majority of the study identified for gestational hypertension was related to screening and risk prediction. Common parameters used for screening were assessment of flow mediated vasodilatation (FMD) of brachial artery, maternal characteristics including biophysical and biochemical parameters, urine creatinine level, and fetal characteristics. Measurement of FMD of the brachial artery was mentioned in two papers included in this review. Both studies recommended FMD to be a potentially effective tool for predicting hypertension during pregnancy at the earliest. A reduction of FMD in those who developed hypertension and further reduction by an average of 2.6% was observed in the 3^rd^ trimester. Mean ± SD Intervals between measurements of FMD and development of PIH were 13.8±2.9 (9.1_Min_-18.4_Max_) weeks for FMD1 and 3.8±3.2 (-1.1_Min_-9.1_Max_) weeks for FMD2 indicating that FMD1 can predict PIH much earlier. About 62% of those with FMD <9% and 16% for those with FMD of >10% developed PIH [30]. Similarly, another study reported that FMD significantly decreased from the 1st to 3rd trimester. FMD at the 3rd trimester effectively predicts the development of the condition with reported OR = 1.303; 95% CI 1.088–1.562; p = 0.004 with good diagnostic accuracy [31]. Assessing maternal characteristics including BMI, MAP, Age, and Parity, biochemical and biophysical markers, fetal characteristics were reported in eight studies. These parameters, when combined, were reported to be effective in predicting the risk of hypertension at the earliest. Investigation on the association of the combined value of serum β-human chorionic gonadotropin (βhCG) levels and serum lipids during 14 to 20 weeks of pregnancy shows that prevalence of PIH/PE increases with an increase in serum βhCG and 95% of women with PIH/PE shows a significantly higher serum βhCG level of ≥40,000. Similarly, serum total cholesterol, mean triglyceride, and LDL were significantly higher among those who developed PIH/PE [32]. Value of pregnancy-associated plasma protein A (PAPP-A), free βhCG, maternal age, BMI, parity, mean arterial pressure (MAP), and uterine-artery Doppler findings to predict PIH in low risk pregnancy between 11 and 13 weeks plus six days of pregnancy. Participants were followed till delivery, and 9.7% (198) becomes hypertensive (Case cohort) while 80.9% (1651) remained normotensive (Control cohort). The case versus control cohort comparisons of maternal, fetal, and neonatal characteristics shows a significant difference in BMI, mean Ut-PI, mean PAPP-A serum concentration, birth weight and also the presence of early diastolic notch was greater in the case-cohort group (P = 0.02), and a combined analysis of diagnostic accuracy produced a good sensitivity and specificity [33]. A similar investigation calculated the risk for early or late onset pre-eclampsia using biochemical markers (serum PLGF and PAPPA), maternal characteristics (age, parity, BMI, medical and pregnancy history), MAP, Mean Ut-PI, crown rump length (CRL), and gestational age (GA). About 64% of cases with a median age of 33.2 (36–31) years and BMI 27.0 (23.7–30.3) kg/m^2^ reported a high risk (1:6) for both early (n = 4) and late PE (n = 23). The age of the mother was observed to have a significant effect in the high-risk groups (p < 0.001), corroborated by the findings that all screen positive cases were in the age group between 31–36 years. Similarly, 58% of women with a previous history of HT reported a significant reduction in PLGF and PAPP-A with a significant increase (p < 0.001) in the test risk (1:8) for late-onset PE. A significant Multiple of Median (MoM) deviation in Ut-PI (19%), PAPP-A (31%), and PGLF (24%) was observed in high-risk groups with a history of PE and high-risk group with a history of hypertension, only PAPP-A (49%) and PLGF (30%) reported having a significant MoM deviation to the control group. A significant decrease of MoMs (PAPP-A and PLGF) was observed in 70–96% of cases in the high-risk groups. When using the data for pregnancy outcome; the overall sensitivity and specificity of the screening test for detecting both early and late onset PE was 83% and 97.6%, respectively. However, for detecting early onset PE, the sensitivity and specificity of the screening test was 44.4% and 100% with a reported accuracy of 99.2%, whereas for detecting late onset sensitivity and specificity was 93.8% and 97.7% with a reported accuracy of 97.5%. NPV was more than 100% for both early and late onset PE [34]. Combined clinical (MAP), biophysical (Ut-PI), and biochemical (PLGF) model for predicting PE in the late 1^st^ trimester had better performance compared to PLGF alone in detecting pre-eclampsia and gestational hypertension [35].

Soluble tumour necrosis factor-like weak inducer of apoptosis (sTWEAK) which is a multipurpose cytokine linked with endothelial dysfunction; a key process in pre-eclampsia shows a significant reduction from baseline to follow up (4.03±0.37 to 1.93±0.23 mg/dl) and is significantly lower when compared to normotensive high risk and negative control group (p<0.001). Similarly, the high-risk group that developed PE/PIH has a significantly lower PLGF value than normotensive high risk and negative control groups (p = 0.013). Pregnant women with lower sTWEAK levels at baseline and follow up developed PE/PIH towards the end of pregnancy, indicating its link to PE pathogenesis [36]. Assessment of Fetal specific marker hypermethylated RASSF1A gene to quantify cffDNA and its relationship with placental protein-13 (pp-13), PAPP-A and urine proteins in diagnosed pre-eclamptic cases show that the mean hypermethylated RASSF1A concentrations, pp-13, PAPP-A and maternal urine protein were significantly higher among the preeclampsia cases in each group of gestation as compared to controls (P < 0.001). The mean concentrations of pp-13 and PAPP-A showed an increasing pattern with gestational age (from 21 weeks to 40 weeks). The concentration of protein in urine varied from 43 to 240 mg/24 h in controls and 315.68 to 886.16 mg/24 h in cases and a multivariate Pearson’s correlation shows that both pp-13 (r = 0.697, P < 0.001) and PAPP-A (r = 0.833, P < 0.001) shows a strong and significant positive correlation with hypermethylated RASSF1A. Hypermethylated RASSF1A was also highly correlated (r = 0.797, P < 0.001) with the urine protein values. When the ROC curve was drawn, the optimum cut-off values of hypermethylated RASSF1A in each gestational group gives sensitivity and specificity that ranges from 85–100% with a high PPV and NPV indicating the reliability of the approach for pre-eclampsia diagnosis [37]. Uric acid as a predictor for severity in PIH shows that serum uric acid was significantly higher in the PIH group than the normotensive group (5.46 ± 1.51 vs. 4.03 ± 0.69, p = 0.001). Receptor Operating Characteristics curve (ROC) curve was drawn to determine the diagnostic utility of serum uric acid, and a Sensitivity/Specificity of 79.07%71.19% were reported with AUC = 0.805. thus, this study concluded that uric acid assessment is a convenient and efficient method for early prediction of PIH [38]. Microalbuminuria at mid-pregnancy using the Albumin and creatinine ratio (ACR) as a predictor of pre-eclampsia between 24–28 GA was evaluated, and ACR between 30 and 299 mg/g creatinine was considered as Microalbuminuria. Of the 144 pregnant women tested, 22.9% microalbuminuria was observed, and of these, 36% developed PE. Mean ACR was significantly higher among severe PE cases. The study also reported the accuracy of ACR for predicting PE with a sensitivity of 92.3%, a specificity of 83.9%, a PPV of was 36.7%, and an NPV of 99.09%. Thus Concluded microalbuminuria can potentially be an effective predictor for pre-eclampsia, and ACR is the optimal tool for detecting microalbuminuria [39].

### Common approach to management

Management modalities were reported in seven studies, but of the seven studies, two measured the efficacy and safety of treatment during pregnancy and two related to management during labour. A retrospective evaluation of the efficacy and safety of administering antihypertensive drugs in GH cases admitted to a tertiary care hospital shows that monotherapy with Nifedipine was reported to be the most commonly used anti-hypertensive drugs administered in 49.5%, and the least was a monotherapy of Methyldopa, which was administered in only 3.5% of the cases. A dual therapy of Nifedipine and labetalol was administered in 36%, and Nifedipine plus Methyldopa was administered in 8% of the cases. Triple therapy of Nifedipine, Methyldopa, and Labetalol was also administered in 4% of the cases. About 51.5% of the cases achieved controlled blood pressure with monotherapy, 39.5% with dual drug therapy, and 3% with triple drug therapy. About 6% of the cases remained unresponsive to any of the drugs. The percentage of preterm, full-term, and late-term deliveries were 19.5%, 22.5%; and 58%, respectively [40]. Similarly, calcium channel blockers, Nifedipine, β-blockers, atenolol, alpha methyldopa (Sympatholytics), furosemide (diuretics), and nitrates were the preferred drugs of choice, and all eclampsia cases received Magnesium Sulphate (MgSO_4_), but the same was prescribed in only 5.8% of the pre-eclampsia cases. Phenytoin was the drug of choice for those with adverse reactions to MgSO_4_. However, the study criticised the high prescription rate of rapid-acting sublingual Nifedipine; given the fact that it is associated with adverse maternal and foetal outcome. Also the fact that labetalol, which is known for its efficacy and safety in treating acute PIH, was prescribed in a very small proportion of cases [41]. A retrospective analysis of case records of 5991 eclamptic patients admitted to the tertiary care hospital from Jan 1999 to Dec 2008. It analyzed the management protocol throughout these years and the maternal-fetal outcome recorded. Data from 1999–2000 reported 50% of cases were subjected to the lytic cocktail (Menon’s regime), and 50% were managed with diazepam. Between the years 2001–2002, all cases were subjected to phenytoin sodium, whereas from 2003–2004 out of 1044 cases, only 10% (104) received Phenytoin Sodium Injection while the remaining 90% (940) were injected with MgSO4. Between 2005–2008 all 2352 eclamptic cases were exposed to MgSO4 injection. Recurrence of convulsion was reported to be the lowest among those who were subjected to inj. magnesium sulphate (5.7%). Those subjected to diazepam were reported to have the highest mean induction delivery interval, 22.23±3.2 hrs. While the lowest was 12.75±2.4 hrs. reported among those who were injected with phenytoin sodium. Prevalence caesarean section was highest among those treated with MgSO4 and phenytoin sodium. Perinatal mortality was also highest in the year 2001–2002 (32.5%) when Phenytoin sodium alone was used as an anticonvulsant and lowest in the year 2005–2008 (22.4%) when only injection MgSO4 was used. Prevalence of LBW was also low during the year 2005–2008 and 2003–2004 [42]. Keepanasseril 2017 [43] compared the efficacy of two drug regimens, the Dhaka regime vs. the loading dose only regime as prophylaxis for seizures in severe pre-eclamptic cases. The low-dose Dhaka regime (Group A, n = 201) were subjected to a loading dose of 10 g of MgSO_4_; 4 g as 20% IV infusion over 10–15 minutes and 3 g as a 50% solution given Intramuscularly in each buttock and 2.5g as 50% solution was given every four hours and continued till 24 hours of postpartum was also administered in alternate buttocks as a maintenance dose. The ‘loading dose only’ (Group B, n = 201) received 10 g of MgSO_4_; 4 g as 20% IV infusion over 10–15 minutes and 6 g as 50% solution intramuscularly 3 g in each buttock. All participants, irrespective of the groups, were allocated to an additional IV dose of 2 g MgSO_4_ as 20% solution and 2.5 g given every four hours and continued till 24 hours of postpartum or 24 hours after the last convulsion. The study reported that 2.24% of women on prophylactic MgSO4 developed eclampsia, 1.49% in the low-dose Dhaka group, and 2.98% in the loading dose only regime group. Mean birth weight of the babies was significantly lower in the ‘low dose Dhaka’ regime than those when compared with the ‘loading dose only’ regime (1895.5 ± 49.5 g vs. 2053.0 ± 53.2 g, p = 0.031) group indicating that a single loading dose only regime may be an effective alternative to the standard 24-hour therapy.

Two studies compared the effectiveness of transcervical Foley catheter and oral misoprostol tablets to induce labour among hypertensive women in India. Women who sufficed the eligibility criteria were randomized to induce labour either by using a transcervical Foley catheter (silicone, size 18F with 30-ml balloon) or 25 mcg of oral misoprostol tablets administered at every 2 hours’ interval for a maximum of 12 doses or until active labour commenced. Dosage was increased to 50 mcg if contractions fail to commenced after two doses in primigravida women. The findings show that vaginal delivery within 24 hours of induction was higher in the oral misoprostol group. Vaginal delivery was also reported to be higher in the oral misoprostol group (59.3%) as compared to the Foley catheter group (49.8%), and the difference is statistically significant (P = 0.0210). The time interval from randomization to induction commencement was four times higher for the Foley group. A reduction of 90 minutes from induction to delivery was observed in the oral misoprostol group. The findings reflected that oral misoprostol improves rates of vaginal delivery within 24 hours of induction (Leigh et al., 2018) [44]. Another randomized controlled trial (INFORM) [45] was conducted in 2 public hospitals in Nagpur, India, to compare relative risk between oral misoprostol and transcervical Foley catheterization for induction of labour in hypertensive pregnancy. The study reported that oral misoprostol of 25 μg every 2 h for a maximum of 12 doses were better in achieving vaginal delivery within 24 hours than transcervical Foley catheter. The reported mean time from the start of induction to birth was 771 min for the oral misoprostol group and 861 min for the Foley catheter group. About 52% of the women in the oral misoprostol group require oxytocin, and 81·6% in the Foley catheter group. Thus, low-dose misoprostol (25 μg) can potentially be safe and effective for induction of labour for mild hypertensive pregnancy in a low resource setting. The study also reported the challenges of managing in the low resource setup; unavailability of infusion pumps is one and fluid overload due to IV oxytocin.

### Gestational hypothyroidism

There is an extreme dearth of good quality evidence related to GH in India. The four studies identified in this review reported only prevalence and maternal-fetal outcome; only one study mentioned treatment options for overt and subclinical hypothyroidism. Routine screening persists to be a challenge in India, and gestational thyroid function screening was recommended only for high-risk pregnancies with past relevant medical and family history. On the contrary, studies included in this review reported a considerably high prevalence of overt and subclinical hypothyroidism with associated adverse perinatal outcomes indicating the need for a routine thyroid function screening for all pregnant women. Current practise guidelines recommend a high risk based screening and treatment with levothyroxine (LT4) supplementation for pregnant women with overt or mild hypothyroidism, defined by an asymptomatic serum TSH level more than 2.5 mIU/L [46]. Diagnosis of subclinical hypothyroidism at TSH values between 5–10 mU/L yields a prevalence of 2.8%. However, when the cut-off is reduced to <2.3 mU/L, the prevalence increases to 19%. TPO antibody was positive among 57.1% of women with hypothyroidism (p = 0.012) and 7.1% in euthyroid women. The study also reported a significant increase in the prevalence of hypothyroidism with increased gestational age [47]. Participants with TSH <2 μIU/mL and TPOAb negative were considered as normal, and participants with elevated TSH (TSH 2–4 μIU/mL or TSH > four μIU/mL) and TPOAb positive were considered as abnormal and were treated with thyroxine to maintain TSH in the range of 0.2–2 μIU/mL. TSH was repeated at six weeks, once when the treatment was initiated or changed, and once the TSH became normal, it was repeated twice monthly. TPOAb titers of participants with TPOAb positive during the initial testing were monitored every trimester. The prevalence of hypothyroidism recorded was 4.8%. Pregnant women with TSH 2–4 μIU/mL without TAI were reported to have significantly increased TSH (by 0.25 μIU/mL; P = 0.029); decrease in FT3/FT4 (P = 0.025 and 0.033, resp.) at 6 months of pregnancy; 5.1% (2) had TSH >4 μIU/mL at 6 month follow up; and one stillbirth. Pregnant women with TSH > 4 μIU/mL were reported to be older as compared to other groups (P = 0.02), and the rate of miscarriage was three times higher in patients with hypothyroidism (P = 0.02); 3 patients were overtly hypothyroid (TSH > 10 μIU/mL). Pregnant women with TSH 2–4 μIU/mL with TAI were reported to have been treated with thyroxine with regular monitoring that led to the significant decline in FT3 and FT4 at the late half of the pregnancy. In this study, 40% of the hypothyroid and 45% of TPOAb positive cases did not report any of the known high-risk characteristics, indicating that about 60% of the hypothyroid or TPOAb positive cases could have been missed by targeted case finding, hence a universal screening is recommended [48]. Similarly, when TSH estimation values above 5.5 mIU/L or below 0.5 mIU/L were considered as abnormal, and those with abnormal TSH values were subjected to free T4 estimation. Women with high TSH and low-free T4 (overt hypothyroidism) were further subjected to thyroid peroxidase antibodies (TPO-Ab) estimation. The prevalence of overt hypothyroid, subclinical hypothyroid, overt hyperthyroid, and subclinical hyperthyroid were 4.58%, 6.47%, 0.78%, and 0.94%, respectively. The women among the overt hypothyroid group have a statistically significant tendency to develop pregnancy-induced hypertension, IUGR, and intrauterine fetal demise. A statistically significant number of pregnant women with subclinical hypothyroidism underwent caesarian section due to fetal distress [49]. Ajmani et al., 2014 [50] evaluated thyroid dysfunction in normal pregnancy and the impact on maternal and fetal outcome following trimester specific TSH value criteria guidelines given by the American thyroid association in 2011. Participants were diagnosed with Subclinical hypothyroid if TSH >3.0 μIU/l and Overt were hypothyroid if TSH >3.0 μIU/l with low Free T4 <0.8 ng/dl). Thyroid dysfunctions were treated, and tests were repeated every six weeks. Patients were monitored and followed until the end of pregnancy. The overall prevalence of overt hypothyroidism was 25%, and subclinical hypothyroidism was 75%. The positive anti-TPO antibody was reported in 52% of the hypothyroid cases but none in hyperthyroid patients. Subclinical hypothyroidism was significantly associated with preeclampsia and placental abruption, and the cesarean section rate was significantly higher in patients with overt hypothyroidism. Adverse fetal outcomes like spontaneous abortion, preterm birth, low birth weight, and intrauterine growth retardation were also significantly higher in subclinical and overt hypothyroidism. Few important findings from this study like a high maternal and adverse fetal outcome among subclinical and overt hypothyroidism and also the fact that prevalence of subclinical cases was the highest indicates the need for universal screening for improving pregnancy outcome.

## Discussion

Most pregnant women are at risk of developing pregnancy induced illness anytime during pregnancy or during childbirth that calls for immediate skilled intervention. Among many others, GDM, HIP, and GH are a few of the high-risk conditions that can complicate pregnancy and increase the risk of adverse maternal and foetal outcomes. Global and national morbidity and mortality related to this are substantial and significant. However, still, a uniform approach to diagnosis and management of these disorders during pregnancy persists in being challenged in many developing countries. Owing to this, all pregnancies should be subjected to routine screening through regular ANC visits provided by trained health personnel for identifying high-risk cases.

### Gestational diabetes

The most common screening and diagnostic approach reported is OGTT of 75 g, followed by 80-100g of OGTT and PBG estimation at fasting and 1 hour and 2 hours. As seen from this review, glucose load for screening, blood glucose estimation time, cut-off values, and diagnosis criteria were heterogamous. ACOG guidelines recommended universal screening using the 2-step procedure by assessing risk factors, or 50-g, 1-hour glucose test followed 100-g, 3-hour OGTT among with positive initial screening result but researchers have also found out that a 1-step procedure using 75-g, 2-hour test following IADPSG single-step criteria increase the detection rate by 2–3 times that of the 2-step procedure. However, many recent publications recommended a definitive diagnosis by OGTT for those positive during the initial screening [51]. Two steps procedures using the Australian Diabetes in Pregnancy Society criteria were also reported to be effective with high diagnostic accuracy and reported that OGTT for positive GCT cases yields a high sensitivity (80%) and specificity (90%) [52]. However, Deveer, 2013 [53] questioned this two-step approach and suggested the need for further exploration and revision of the approach. A study from Saudi Arabia [54] reported 50g GCT in non-fasting followed by 100g OGTT for those positive with 50g GCT give a diagnostic yield of 35% and a post 50g GCT load cut off value of 7.8 mmol/L gave the maximum sensitivity and specificity that is 88% and 84% respectively along with 82% PPV. However, a 100% PPV cut off obtained at PBG level >10.4 mmol/L. Thus, screening 24–28 weeks with 50g GCT is reliable with a cut off of 7.8 mmol/L 1-hour PBG. Rajput et al., 2012 [24] evaluated the efficacy of HbA1c in combination with OGTT using criteria ADA and IADPSG criteria and reported good sensitivity and specificity at various cut-offs. Similarly, Khan 2019 [25] evaluated glucose tolerance patterns of pregnant women subjected to 2-H OGTT for comparing fasting, 1-H, 2-H post-glucose load results, HbA1c and reported that a combined glucose estimation of these values produced an AUC of 0.962 (95% CI: 0.935–0.988). However, HbA1c as single parameters yields a comparatively low diagnostic accuracy. Risk factors approach for screening and diagnosis was reported in two studies, but findings were inconclusive, and both papers recommended universal screening [22, 23].

Administration of 500mg metformin 15 minutes before breakfast among those with ≥2 abnormal PBG values; FBG >95 mg/dl and 2hour value >120 mg/dl post-MNT found to be effective in controlling blood sugar in 93.8% (60) of cases and only <2% required insulin therapy [26]. A Structured model of care (MOC)- which included MNT, regular physical activity, intensive counselling, and regular blood glucose monitoring significantly reduced the proportion of cases required insulin therapy, indicating that management of GDM with MNT and focused counselling can be effective in minimizing the need for insulin therapy and in bringing down maternal and neonatal complication [27]. The common form of treatment of GDM in India was insulin therapy, and oral metformin was the most commonly prescribed agent [55]. Blood glucose monitoring, as evident from this review, is a crucial process for monitoring prognosis and for deciding the line of treatment for the cases. Findings from this review are in line with other existing evidence reported elsewhere; Chen 2003 [56] compared two methods for daily glycaemic profile monitoring, i.e., continuous vs. self-monitoring; along with the evaluation of different treatment strategies and concluded that continuous glucose monitoring was more effective in monitoring GDM cases as well as for bringing adjustments in diabetes therapy. The study also revealed that high PPBG levels were more accurately detected by continuous glucose monitoring, thereby reduced the chance of missing the undetected nocturnal hypoglycaemic events. MNT was the initial approach to management, and cases were subjected to either oral metformin or Insulin only if the desired blood glucose level cannot be controlled after two weeks of MNT. Similarly, Diet modifications, physical activities, self-glucose monitoring, and insulin therapy was the most common management with MNT and exercises reported as first line treatment [57, 58]. This shows that the MNT was effective in glycaemic control with adequate weight gain and in improving pregnancy outcomes and that Metformin may be a safer and effective oral hypoglycaemic agent, which can be useful in a limited resource setting.

### Gestational hypertension

Predicting PIH at the earliest is an essential preventive strategy for minimizing maternal and foetal complications. FMD as an approach to predicting GH/PE was reported in two studies. Both reported FMD as an effective non-invasive approach [30, 31], which is consistent with a recent review which reported that FMD is lower among women who later developed preeclampsia when compared to those without preeclampsia [59] and when taking ≥3.0% as a normal cut-off value for FMD in the brachial artery, the PPV for developing PE was 90%, and NPV was 100% which adds evidence to the assumption that women with impaired FMD in the brachial artery during early pregnancy are at risk for developing pre-eclampsia [60]. Maternal characteristics; age, parity, BMI, biochemical markers; serum PLGF, PAPP-A, βhCG, Doppler studies; MAP, Ut-PI, CRL, and foetal specific markers were also reported to be effective in predicting the development of hypertension/pre-eclampsia [32–35, 37] Conversely, transvaginal colour Doppler assessment of the uterine arteries at 23 weeks of gestation reported a one-stage colour Doppler screening program at 23 weeks identifies most women who subsequently develop severe pre-eclampsia and/or foetal growth restriction [61]. In line with these findings, Akolekar et al., 2013 [62] constructed a model for the prediction of preeclampsia based on maternal characteristics, biophysical and biochemical markers at 11–13 weeks of gestation. They reported that screening by maternal characteristics, biophysical and biochemical markers detected 96% of cases of PE requiring delivery before 34 weeks and 54% of all cases of PE at a fixed false-positive rate of 10%. One study [39] evaluated microalbuminuria at mid-pregnancy using the ACR as a predictor of PE and observed that mean ACR was significantly higher among severe PE cases and ACR for predicting PE shows a sensitivity of 92.3%, a specificity of 83.9%, PPV of 36.7% and NPV of 99.09%. This finding is similar to a report by Zen et al. 2019 [63], that a high level of uACR in the third trimester was associated with the development of pre-eclampsia (p = 0.01) and Rodriguez et al. 1998 [64], which reported that 83% of patients with microalbuminuria level ≥11 μg/ml and calcium/creatinine ratio ≤0.04 developed pre-eclampsia whereas 94% of women with optimum microalbuminuria and calcium/creatinine ratio did not. Serum Uric Acid is also a convenient and efficient method for early prediction of PIH with a reported sensitivity and specificity of 79% and 71%, respectively [38]. Investigation of sTWEAK and its prognostic ability in high-risk pregnancy revealed that sTWEAK subsequently reduced from 4.03±0.37 ng/dl at baseline to 1.93±0.23 mg/dl at follow up and is significantly lower when compared to normotensive high-risk and negative control group (p<0.001). The high-risk group that developed PE/PIH also reported having a significantly lower PLGF value, which strengthened the hypothesis that low sTWEAK is related to the progression of disease severity, indicating the potential of sTWEAK in identifying high-risk cases at the earliest [36]. A similar finding was reported by Yildirim et al. 2016, in which sTWEAK levels were significantly lower in pre-eclamptic patients (332 ± 144 pg/mL) than in control subjects (412 ± 166 pg/mL) (p = 0.04). However, the authors warrant further investigation to build evidence on the role of sTWEAK as a predictor for pre-eclampsia [65].

Five studies reported on the management of hypertension in pregnancy. One paper discussed only related to management during delivery, three talked about antihypertensive drugs and anti-convulsant for pre-eclampsia and eclampsia, and one study discussed referral systems and their impact on the management of eclampsia patients. Overall findings show MgSO4 as the most common anticonvulsant for prevention and treatment of eclampsia and Nifedipine as the most common anti-hypertensive agents [40, 42]. However, a high prescription rate of rapid acting Nifedipine was strongly criticized and suggested a periodic review of the treatment protocols to improve pregnancy outcome [41]. A single loading dose, the only regime, may be an effective alternative to the standard 24-hour therapy for seizure prevention in severe preeclampsia cases [43]. A systematic review by Duley 2006 [66] shows similar findings; Magnesium sulphate, when administered to pre-eclamptic cases, reduced the risk of eclampsia to more than half, and for those with eclampsia, Magnesium Sulphate is the first choice anticonvulsant followed by diazepam, phenytoin, and lytic cocktail. Lin 2018 [67] reviewed the choice and use of drugs for the management of pregnancy induced hypertension. The first line drug commonly prescribed for managing primary hypertension were oral methyldopa, labetalol, and Nifedipine. Appropriate drugs of choice for emergency treatment are Intravenous labetalol, Intravenous hydralazine, and Sublingual Nifedipine. Appropriate management during delivery is essential to ensure mother and neonate safety; Leigh 2018 [44] compared trans-cervical Foley catheter to oral misoprostol tablets for inducing labour in hypertensive women and reported that Oral misoprostol improves rates of vaginal delivery within 24 hrs of induction and INFORM trial also reported that Oral Misoprostol of 25 μg every two hours for a maximum of 12 doses resulted in a mean interval time of 771 min (from the start of induction to birth) as compared to 861 min for the Foley catheter group. Vaginal delivery was also reported to be higher in the misoprostol (57·0% vs. 47·0%; absolute risk difference 10·0%, 95% CI 2·0–17·9) when compared to the Foley catheter group and a little over than half (52%) of the women in the oral misoprostol group requires oxytocin whereas 81·6% of those in the Foley catheter group required oxytocin [45]. Findings from another country; PROBAT-II study by Eikelder et al. [68], shows no significant differences in maternal and neonatal outcome between oral Misoprostol and Foley catheter for induction of labour but reported fewer caesarean section in the oral misoprostol group, which was also reported in the INFORM trial. Conversely, a review by Eikelder et al., 2016 [69] reported that a Foley catheter, compared to Oral Misoprostol, shows less hyper-stimulation and is associated with instrumental deliveries, fewer caesarean deliveries for those with non-reassuring foetal heart rate but no differences in neonatal outcomes were reported. Additionally, appropriate referral services and hospital networking facilitates effective management of emergency obstetrics cases like eclampsia and minimized delay in access to treatment [70].

### Gestational hypothyroidism

Routine screening for detecting thyroid dysfunction during pregnancy persists to be a challenge in India, and gestational thyroid function screening was recommended only for high-risk pregnancy with past relevant medical and family history. On the contrary, these studies reported a high prevalence of overt and subclinical hypothyroidism with associated adverse perinatal outcomes indicating the need for routine thyroid function screening for all pregnant women or to decrease the threshold for screening. All four articles included in this rapid review reported the screening and diagnostic approach to classifying thyroid dysfunction in pregnancy, and two papers mentioned very briefly about treatment strategy, but none of the paper measured the sensitivity and specificity of the screening and diagnostic tool, nor did they mention the treatment safety and efficacy. The most common screening approach reported in all the articles was a trimester specific Thyroid Stimulating Hormones (TSH) test followed by estimation of Thyroxine (free T4) and Thyroid antibody test (TPO-Ab) [47, 49, 50] and Levothyroxine was the treatment of choice with regular monitoring of TSH level for Overt hypothyroidism. However, a dilemma about whether to treat subclinical hypothyroidism or not persists as there is a lack of evidence suggesting potential benefits. Teng 2013 [71] conducted a review on the diagnosis and treatment of overt and subclinical hypothyroidism in pregnancy. It is recommended that a trimester-specific TSH value should be used while considering iodine sufficiency. The trimester-specific reference range given for iodine sufficient region which doesn't have an existing standard reference is 2·5 mIU/L for the first trimester, 3·0 mU/L for the second, and 3·0–3·5 mIU/L for the third. For the diagnosis of hypothyroidism in pregnancy currently, both serum TSH along with T4 values with trimester-specific ranges should be considered. The report also suggested that women with known hypothyroid during pregnancy should be checked periodically for serum TSH concentration and treated accordingly. For treating pregnant women with overt hypothyroidism, levothyroxine dose would be prescribed to normalise the thyroid functions by estimating the TSH values within 30–40 days from initiation of levothyroxine and then once in every 4–6 weeks. Alexander 2004 [72] conducted a study to understand the exact timing and amount of levothyroxine adjustment required during pregnancy for known hypothyroid patients and reported a 47% increase in the mean levothyroxine requirement from the first half of pregnancy until delivery. The median onset of the increase was noticed at eight weeks of gestation and levothyroxine requirement plateaued by the 16th week. The study proposed a 30% increase in LT4 dose when a woman with hypothyroidism confirmed with pregnancy, and it should be followed by monitoring of serum thyrotropin levels and the proper adjustment in levothyroxine dose. Few important findings from these articles, like a high maternal and adverse fetal outcome among subclinical and overt hypothyroidism, and the fact that the prevalence of subclinical cases was the highest indicates the need for universal screening for improving pregnancy outcome. Thus, the study recommended that screening for hypothyroidism to be included as a part of routine antenatal care assessment to improve maternal and fetal outcomes.

## Conclusion

India has national guidelines for Screening, Diagnosis, and Management of GDM (2018) and GH (2014); however, there is no proper PIH guideline. These guidelines need to be contextualized and modified based on the local population's need for effective treatment and adoption. The findings of this review show that early screening for predicting the risk is an effective deterrent strategy for the prevention, treatment, and management of high-risk pregnancies, thereby curtailing the related morbidities and mortality. However, the definitive diagnosis and medical management protocol for these high-risk pregnancies are found to be heterogeneous. As India enters the third stage of Obstetric transition phenomena [73], the development of evidence-based guidelines for improving knowledge and practice is imperative to minimize maternal and foetal adverse outcomes. Primary research on community-based screening and management of these high-risk conditions during pregnancy is scanty, obviating the possibility for developing tailored community-based management guidelines for these high-risk conditions. Hence, we strongly recommend researchers build on high quality evidence through primary research with an adequate sample size at the facility level and/or community level. Evidence from these studies could form the foundation for developing the country-specific guidelines that can be adopted at various levels of the country's health system.

## Supporting information

S1 File (DOC)

S2 File (DOCX)
