# Supplementary material for: Generating evidence on screening, diagnosis and management of non-communicable diseases during pregnancy; a scoping review of current gap and practice in India with a comparison of Asian context
Source: PLoS One. 2021 Feb 1;16(2):e0244136. doi: 10.1371/journal.pone.0244136 (PMC7850625; doi:10.1371/journal.pone.0244136)
Supplement: S2 File — (DOCX) [file pone.0244136.s002.docx]

**PubMed Search Strings**

| ((((((((((evaluate) OR evaluating) OR evaluation) OR effectiveness) OR impact) OR effect) OR evaluat*)) AND ((((((((((((((((((((((((management) OR treatment) OR therapy) OR guidelines) OR management guidelines) OR treatment guidelines) OR treatment protocol) OR standard protocol) OR therapeutic guideline) OR management protocol) OR standard treatment) OR standard treatment procedure) OR standard treatment protocol) OR treatment delivery) OR high-risk pregnancy care) OR recommendation) OR policy) OR intervention) OR case management) OR supportive therapy) OR supportive care) OR supportive treatment) OR specific therapy) OR specific treatment)) AND (((((((((((((((((((((((gestational diabetes) OR gestational hypertension) OR gestational hypothyroidism) OR pre-eclampsia) OR eclampsia) OR non communicable disease) OR pregnancy induced hypertension) OR pregnancy induced hypothyroidism) OR pregnancy induced diabetes) OR hyperglycemia) OR hypertension) OR pregnancy glycaemic index) OR hypo thyroxin ?emia) OR hypothyroid*) OR thyroid deficien*) OR thyroid dysfunction) OR thyroid Disease*) OR thyroid insufficienc*) OR pregnancy-induced hypertension) OR hypertensi* AND disorder*) OR preeclampsia) OR PIH) OR GDM)) AND ((((((((((((((((pregnant) OR antennal) OR prenatal) OR gestation) OR conception) OR perinatal) OR intranatal) OR postnatal) OR high risk pregnancy) OR high risk pregnancies) OR risk pregnancy) OR high risk delivery) OR risk delivery) OR high-risk pregnancy) OR high-risk pregnancies) OR risk pregnancies) AND ("last 10 years"[PDat] AND Humans[Mesh]) AND ("last 10 years"[PDat] AND Humans[Mesh]) |
| --- |
